# Dental Fear among Medical and Dental Undergraduates

**DOI:** 10.1155/2014/747508

**Published:** 2014-10-16

**Authors:** H. Hakim, I. A. Razak

**Affiliations:** ^1^Faculty of Dentistry, University of Malaya, Malaysia; ^2^Department of Oral Health & Clinical Prevention, Faculty of Dentistry, University of Malaya, 50603 Kuala Lumpur, Malaysia

## Abstract

*Objective*. To assess the prevalence and level of dental fear among health related undergraduates and to identify factors causing such fear using Kleinknecht's Dental Fear Survey (DFS) questionnaire. *Methods*. Kleinknecht's DFS questionnaire was used to assess dental fear and anxiety among the entire enrollment of the medical and dental undergraduates' of the University of Malaya. *Results*. Overall response rate was 82.2%. Dental students reported higher prevalence of dental fear (96.0% versus 90.4%). However, most of the fear encountered among dental students was in the low fear category as compared to their medical counterpart (69.2 versus 51.2%). Significantly more medical students cancelled dental appointment due to fear compared to dental students (*P* = 0.004). “Heart beats faster” and “muscle being tensed” were the top two physiological responses experienced by the respondents. “Drill” and “anesthetic needle” were the most fear provoking objects among respondents of both faculties. *Conclusion*. Dental fear and anxiety are a common problem encountered among medical and dental undergraduates who represent future health care professionals. Also, high level of dental fear and anxiety leads to the avoidance of the dental services.

## 1. Introduction

Behavioral sciences have become an increasingly important component in dental education and research. One aspect of it involves the application of psychological methods to the study of behaviour and attitudes relevant to health, illness, and health care, particularly about being anxious or fearful of dentists and dentistry as well as of dental pain [1, 2].

Anxiety is defined as an intuitive, vague, unpleasant feeling that something undesirable is going to happen, while fear is the anticipation of threat obtained by an identifiable source [3]. However, the terms dental fear and anxiety are often used interchangeably.

Dentistry has advanced throughout the years with many improved technologies and better understanding of patient's need. Yet these improvements have not been able to eliminate or substantially reduce dental fear and anxiety among patients. Studies have shown that restorative procedures, namely, the sight, sound, and vibrational sensation of rotary, dental drills and sight and sensation of a dental local anesthetic injection, are the most potent causes of dental fear and anxiety [4]. The drill and anesthetic needle were also identified as the most fear provoking objects among Malaysian antenatal mothers [5]. Studies have also revealed that uninvolved and noninteractive behavior of the dentist towards patients also provokes fear [6].

The prevalence of dental fear and anxiety has been studied in different populations and in different age groups. Most available literatures are from studies undertaken in North American or Northern European populations. Only a few studies have been undertaken on Asian population. Relatively high prevalence has been recorded in US, Australian, Japanese, Swedish, Danish, French, Finish, and Singaporean studies [7–14] ranging from 10 to 50%. Similarly, the prevalence of dental fear and anxiety in Malaysia has been found to be high. A study on dental fear and anxiety among young adult trainee teachers found a 99% prevalence [15]. Studies on 16-year-old adolescents [16] and 10–12-year-old school children [17] showed high prevalence of 98.2% and 81.9%, respectively. A recent study by Savithri [2] on dental fear and utilization behavior among antenatal mothers in Malaysia found a prevalence of up to 94.1%, with 26.5% of them being moderately fearful.

Dental fear and anxiety affect not only general adult population but also university students who are going to become health professionals. A study done by W. M. Al-Omari and M. K. Al-Omari [18] on dental anxiety and factors arousing the anxiety and fear among medical and dental students found that medical students have more dental fear than dental students. Serra-Negra et al., [19] in their study done on Brazilian dental students, found that 27.5% of dental students reported fear when they become dental patients.

This study aims to provide information about dental fear and anxiety among medical and dental undergraduates in Malaysia, their level of fear, and the reasons for dental avoidance.

## 2. Materials and Methods

### 2.1. Study Population

This is a pilot study on dental fear among potential health care professionals in Malaysia. It is cross-sectional and quantitative in design and is undertaken at the University of Malaya which is the oldest and largest public university in Malaysia. There are 34 medical schools (11 public, 24 private) and 12 dental schools (6 public, 6 private) in Malaysia. As this is a pilot study involving one medical and one dental school in Malaysia, the population of this study was the entire enrollment of the medical (*N* = 994) and dental (*N* = 376) undergraduates of the University of Malaya in the 2011/2012 academic year. The age range of the undergraduates was 19–23 years.

### 2.2. Questionnaire

A self-administered Kleinknecht's Dental Fear Survey (DFS) questionnaire was used in this study. This questionnaire has been validated for Southeast Asian countries [20] and has also been used for the Malaysian population [2]. The questionnaire was in English as the study population medium of education is English. The questionnaire was pretested on undergraduates of the Faculty of Dentistry, Universiti Teknologi MARA, Malaysia, and was found to be clear and comprehensible.

Approval to conduct the study was sought from the Deans of the Medical and the Dental Faculties of University of Malaya. The questionnaire was administered in the classroom setting after their lectures. The respondents were informed that their contributions are confidential and entirely voluntary.

The questionnaire consisted of five sections. Section A consists of the participant's demographics: their gender, ethnicity, and family household income. Section B relates to the symptoms of fear respondents experienced when making an appointment. Section C asks respondents to rate themselves about how they feel when dental work is being undertaken. Section D asks respondents to rate themselves regarding fear, anxiety, and unpleasantness during dental treatment.

Kleinknecht's Dental Fear Survey instrument relates to sections B-D comprising a total of 20 questions/items. Each question was assessed by a 5-point Likert scale (K-score ranges from 1 to 5, where 1 = never, 2 = once or twice, 3 = a few times, 4 = often, and 5 = always). For each question, where the response has been dichotomised, K-score of 1 indicates no fear whereas K-score > 1 indicates being fearful. The total K-score for the 20 questions ranges from 20 to 100. Based on the total K-scores of the students, they were then categorized according to various levels of dental fear with increasing scores indicating higher level of fear. 20 indicates “no fear,” score of 21–40 was categorized as low fear, score of 41–79 indicates moderate fear, and score of 80–100 indicates high fear.

## 3. Results

Overall 82.2% of the entire student population responded to this questionnaire. The response rate among undergraduates of the medical faculty was 78.3% (779/994), while 92.2% (347/376) of the dental students responded to the survey. The overall K-score was significantly higher (*P* = 0.005) among medical students 38.6 (SD = 1.4) compared to dental students 35.9 (SD = 1.1). Based on sociodemographic characteristics, medical students who were females and Malays as well as those in the income categories of RM 1500-RM3500 and more than RM 3500 had higher K-scores compared to their dental colleagues. About 27% of the medical students indicated that they cancelled or did not appear for dental treatment due to fear as compared to only 18.7% of dental students (*P* = 0.004).

The “heart beats faster” and “muscle being tensed” were the top two physiological responses experienced by the respondents during dental treatment. “Heart beats faster” was found to be significantly higher among the medical respondents as compared to their dental colleagues whilst the reverse trend was evident for “muscle being tensed” experienced during dental treatment (Table 1).

Kleinknecht's score of fear, anxiety, and unpleasantness when confronted with dental activities was assessed in 12 dental situations. The mean average score of these 12 responses between the medical and dental students is shown in Table 2. Overall medical and dental students had similar fearful experience in having dental work done. Generally, the feelings of fear, anxiety, and unpleasantness were more frequently encountered during the treatment procedures. The drill and anesthetic needle invoke the most fear, anxiety, and unpleasantness. Significantly more medical students frequently reported this to be so in respect of the dental drills. In general, the respondents were less fearful towards the nonoperative dental procedures. Among the lowest rated responses among both medical and dental students were “making a dental appointment” and “when approaching the dental clinic.”

The overall K-score was categorized into different levels of fear. The prevalence of fear among both medical and dental students was high (>90%). Interestingly, the overall prevalence of fear among the dental students (96%) was higher than the medical students (90.4%). However, most of the fear encountered among dental students was in the low fear category as compared to their medical counterpart (69.2 versus 51.2%). Almost 40% of medical students had moderate to high fear as compared to only 27% among dental students (Table 3).

## 4. Discussion

Various cut-off points have been used for categorizing the subjects with no, low, and high dental fears in order to determine the prevalence and level of dental fear and anxiety. A high cut-off point will result in a low prevalence while a low cut-off point will result in a high prevalence. In this study, the level of fear among respondents was classified based on previous local studies [2, 15, 16] for ease of comparison. The fear level was categorized as 20 (no fear), 21–40 (low fear), 41–79 (moderate fear), 80, and above (high fear). However, it should be noted that some other studies have a different classification of high and low dental fear based on their mean DFS score. The score above the mean DFS + 1 SD has also been used to reflect high dental fear [7, 14]. Therefore, comparison with other studies should be done with caution.

This study revealed some very interesting findings. Surprisingly more dental students rated their visit to the dentist as “scary” or “very scary.” This can be due to the fact that they may have encountered more traumatic dental experience in the past [19] or that dental students can anticipate much better than their medical colleagues the steps that will be taken in the delivery of dental care and the risk associated with it. If the students feel afraid of the dental treatment themselves, then they may pass on this insecurity and reinforce the dental fear among their patients or those close to them [19].

In this study, when the result is dichotomised between no fear and fearful, 26.7% of the medical students indicated that they cancelled or did not appear for the dental treatment due to fear. This result varies from a local study [2] on antenatal mothers where 14% of them failed to appear for the dental appointment due to fear. This may be due to the fact that, in the Malaysian public healthcare system, the antenatal mothers are frequently exposed to both antenatal and dental care as part of an inbuilt mechanism of appointments. This avoidance behavior is comparable to the findings in other international studies. In the US, 51.2% of adult subjects who were fearful had delayed making appointments and 9.1% failed to appear on scheduled appointment [7]. Similarly, studies in Japanese, Danish, and Swedish populations drew similar conclusions [9–11]. The delay in getting dental work done as reported in 31.5% of the Japanese population [21] is higher than this study.

It appears that avoiding or delaying dental treatment is a common problem worldwide. However, the respondents in this study have lower rates of cancellation of appointment than that found in the US [7] and almost similar ones to that of the Japanese population [22]. This difference can be explained by cultural differences between the countries. In Malaysia, the public dental service is quite established and covers a wide range of dental treatments except for orthodontics and dental surgical procedures where the patient requires an appointment. Similar to other Asian countries like Japan, Malaysians have a strong sense of respect for people in authority. Once an appointment is made, they show a strong commitment to honour it [2]. Still, there is a strong relationship between dental fear and the avoidance of dental treatment [4, 23].

The physiologic response mostly rated by the studied population was “muscle becomes tense” and “heart beats faster,” The “heart beats faster” was also rated the highest in other Malaysian studies [2, 15, 16]. Elsewhere, other studies have similarly found muscle tenseness as being the most frequently rated followed by “the heart beats faster” [21, 24]. Although response in the different physiologic reactions may vary with different populations and cultures as diverse social groups and cultures accept and translate dental treatment differently, some general trend can be observed across cultures and social norms.

Overall, moderate fear was reported among both the medical and dental undergraduate students with regard to the various activities associated with dental care. The most rated response reported was related to the clinical aspect of dental care, that is, “feeling the needle injected,” “feeling the vibrations of the drill,” “hearing the drill,” and “seeing the anesthetic needle.” Similar findings were also reported in other Malaysian and international studies [2, 7, 11, 14–16, 18, 19, 21, 24–26]. Most likely, this can be due to the fact that “drill” and “anesthetic needle” are common clinical procedures that can invoke pain. Kleinknecht et al. [24] reported in their study that patient ascribes the fear and anxiety to the anticipation of pain during dental treatments. In addition, painful and traumatic experience is predictive of dental fear and anxiety [27, 28]. This will result in the development of negative attitude of patient towards dentistry and dental procedures for a very long time. The contribution of pain to dental fear and anxiety is found to be the major cause of avoidance of dental procedures [29] and therefore intervention of dental fear and anxiety management programmes should be introduced.

The respondents of this study were less fearful towards the nonoperative dental procedures. Among the lowest rated responses among respondents of both faculties were “making a dental appointment” and “when approaching the dental clinic.” Similar results were found in other studies where low responses were found for “making a dental appointment” [2, 21, 24]. Different dental procedures invoke different level of fear among individuals. This study suggests that the respondents were more fearful towards the operative procedures than the nonoperative work. The possible reason for this is that the operative dental procedures are directly or bodily applied to them.

Dental fear and anxiety are a major if not common problem encountered in the general population as well as among health care professionals. Most people encountered dental fear and anxiety when having their dental treatments. This study is confined to the physical and psychological aspects of dental fear and anxiety. As behavior of dentists towards their patients may alleviate the stress patients experience and enhance their satisfaction towards dental care, future studies should also look into the dentist-patient interaction to provide a holistic picture of dental fear and anxiety.

Kleinknecht's DFS questionnaire used in this study is comprehensive comprising 20 questions. Nevertheless, it is easy to apply. As it predicts anxiety accurately, its use in clinical practice is recommended [30, 31]. Hence, the instrument can be used routinely by oral health care professionals on their patients to assess their level of dental fear.

Establishing specialized dental fear clinics will also help in managing the dentally anxious patients (including medical and dental students) to alleviate their dental fear and anxiety. Oral health promotion should be the mainstay of activities in these clinics. The first visit should always be a preventive visit. Patients should be encouraged and assisted in improving their oral hygiene practice that will have a positive impact on their future oral health status. For fearful patients, emergency treatment and preventive care can be initiated as this will expose them to less pain and will diminish their fear and anxiety related to subsequent dental treatments.

The limitation of the study was that the questionnaire was originally constructed for the Western population and has been translated into many languages including Bahasa Melayu [2]. For this study, the questionnaire was administered in English as the study population was deemed to be conversant in the language. The additional use of the questionnaire in the Bahasa Melayu language would have added better accuracy especially for students who do not have a good command of English. However, the impact of this can be considered low as the English questionnaire had been pretested in a random group of dental students in all years of study. Secondly, as in any questionnaire survey, the respondents may hide their true feeling and may also have underreported their dental fear, anxiety, and unpleasantness related to seeking and obtaining dental care.

## 5. Conclusion

The overall prevalence of dental fear among respondents was high (>90%). Whilst dental students (96%) exhibited higher prevalence compared to their medical colleagues (90.4%), most of the fear experienced was in the low fear category (dental 69.2% versus medical 51.2%). Significantly more medical students reported cancelling or not appearing for an appointment due to fear compared to dental students (*P* < 0.05). The “heart beats faster” and “muscle being tensed” were the top two physiological responses experienced and the drill and anesthetic needle were the most fear provoking objects reported by the students of both faculties. Dentists who practice empathy and promote preventive visits to patients as well as the introduction of specialized dental fear clinics for the most dentally anxious patients will reduce fear and anxiety towards dental care.

## Figures and Tables

**Table 1:** Kleinknecht's score of physiologic responses during dental treatment.

Response	Medical *n* = 779		Dental *n* = 347		*P* value
	Mean (SD)	Med (IOR)	Mean (SD)	Med (IQR)	
My muscles become tense	1.88 (0.89)	2.0 (1)	1.99 (0.88)	2.0 (1)	0.027
My breathing rate increases	1.85 (0.90)	2.0 (1)	1.72 (0.85)	1.0 (1)	0.030
I perspire	1.53 (0.81)	1.0 (1)	1.50 (0.77)	1.0 (1)	0.616
I feel nauseated and sick to my stomach	1.48 (0.79)	1.0 (1)	1.27 (0.60)	1.0 (0)	0.000
My heart beats faster	2.07 (0.99)	2.0 (2)	1.83 (0.86)	2.0 (1)	0.000

**Table 2:** Kleinknecht's score of fear, anxiety, and unpleasantness when confronted with dental activities.

Response	Medical *n* = 779		Dental *n* = 347		*P* value
	Mean (SD)	Med (IOR)	Mean (SD)	Med (IQR)	
Making a dental appointment	1.55 (0.84)	1.0 (1)	1.49 (0.77)	1.0 (1)	0.366
Approaching the dental clinic	1.52 (0.80)	1.0 (1)	1.51 (0.81)	1.0 (1)	0.925
Sitting in the waiting room	1.73 (0.95)	1.0 (1)	1.67 (0.85)	1.0 (1)	0.805
Being seated in the dental surgery	2.00 (1.01)	2.0 (2)	2.18 (1.02)	2.0 (2)	0.003
The smell of the surgery	1.79 (0.98)	1.0 (1)	1.68 (0.86)	1.0 (1)	0.180
Seeing the dentist walk in	1.62 (0.87)	1.0 (1)	1.54 (0.79)	1.0 (1)	0.200
Seeing the anesthetic needle	2.42 (1.18)	2.0 (2)	2.44 (1.11)	2.0 (1)	0.623
Feeling the needle injected	2.64 (1.23)	2.0 (2)	2.48 (1.10)	2.0 (1)	0.073
Seeing the drill	2.48 (1.23)	2.0 (2)	2.03 (1.05)	2.0 (2)	0.000
Hearing the drill	2.53 (1.24)	2.0 (2)	2.09 (1.06)	2.0 (2)	0.000
Feeling the vibrations of the drill	2.59 (1.25)	2.0 (2)	2.18 (1.07)	2.0 (2)	0.000
Having your teeth cleaned	1.82 (1.03)	1.0 (1)	1.57 (0.77)	1.0 (1)	0.002
All things considered, how fearful are you of having dental work done?	2.13 (1.00)	2.0 (2)	2.04 (0.82)	2.0 (0)	0.382

**Table 3:** Respondents' level of fear.

	No fear	Low fear	Mod. fear	High fear	*P* value
Medical	70 (9.0%)	399 (51.2%)	305 (39.2%)	5 (0.6%)	0.000
Dental	14 (4.0%)	240 (69.2%)	93 (26.8%)	0 (0.0%)	
Total	**84** (**7.5%**)	**639** (**56.7%**)	**398** (**35.3%**)	**5** (**0.4%**)	
